# Prevalence of nasopharyngeal *Streptococcus pneumoniae* carriage and resistance to macrolides in the setting of azithromycin mass drug administration: analysis from a cluster-randomised controlled trial in Malawi, 2015–17

**DOI:** 10.1016/S2666-5247(21)00279-2

**Published:** 2022-02

**Authors:** John D Hart, Lyson Samikwa, Harry Meleke, Sarah E Burr, Jen Cornick, Khumbo Kalua, Robin L Bailey

**Affiliations:** a Clinical Research Department, London School of Hygiene & Tropical Medicine, London, UK; b Microbiology Department, University of Malawi College of Medicine, Blantyre, Malawi; c Department of Surgery, University of Malawi College of Medicine, Blantyre, Malawi; d Institute of Infection and Global Health, University of Liverpool, Liverpool, UK; e Blantyre Institute for Community Outreach, Blantyre, Malawi

## Abstract

**Background:**

Azithromycin mass drug administration (MDA) could reduce child mortality. However, macrolide resistance, which has generally been reported to develop after whole-community MDA for trachoma control, is a concern, and it has less commonly been studied in the context of treating children to reduce mortality. Here, we report on macrolide resistance after biannual azithromycin MDA at the Malawi site of the MORDOR study.

**Methods:**

In the MORDOR cluster-randomised trial in Malawi, 30 communities in Mangochi District were randomly selected. Communities were randomly assigned to receive azithromycin or placebo by simple randomisation without stratification. Children aged 1–59 months were administered azithromycin 20 mg/kg or placebo as an oral suspension biannually for a total of four treatments in 2015–17. 1200 children (40 children per community) were randomly selected for nasopharyngeal swabs at baseline, 12 months (6 months after the second treatment visit), and 24 months (6 months after the fourth treatment visit). Samples were processed to culture *Streptococcus pneumoniae*. The primary outcome was the proportion of *S pneumoniae* isolates exhibiting macrolide resistance at 12 months and 24 months, assessed in the intention-to-treat population. The study is registered with ClinicalTrials.gov, NCT02048007.

**Findings:**

At baseline, 3467 (76%) of 4541 eligible children in the azithromycin group and 3107 (72%) of 4308 eligible children in the placebo group were treated. 564 nasopharyngeal swabs were taken from the azithromycin group and 563 from the placebo group, with similar numbers of swabs taken at 12 months and 24 months. In both groups at baseline, carriage of *S pneumoniae* was greater than 85% and the proportion of strains resistant to macrolides was 28%. At the 12-month follow-up, macrolide resistance was higher in the azithromycin group (36·9%, 95% CI 32·5–41·2) than in the placebo group (21·6%, 17·7–25·4; OR 2·26, 95% CI 1·46–3·49; p=0·0002). At 24 months, macrolide resistance remained higher in the azithromycin group (43·9%, 39·2–48·5) compared with placebo (32·8%, 28·5–37·1; OR 1·66, 1·15–2·40; p=0·0069).

**Interpretation:**

These findings support previous evidence from trachoma MDA programmes and suggest that monitoring of macrolide resistance should remain a key component of azithromycin interventions for reducing child mortality.

**Funding:**

Bill & Melinda Gates Foundation.

## Introduction

Mass drug administration (MDA) with azithromycin is an effective strategy for the control of blinding trachoma caused by ocular *Chlamydia trachomatis* infection.^1^, ^2^, ^3^, ^4^ Azithromycin is an appealing MDA candidate due to its safety profile, long duration of action, and the availability of low-cost generic formulations.^5^, ^6^, ^7^ More than one billion doses have been distributed as part of the WHO SAFE strategy for the elimination of trachoma.^8^ In addition, azithromycin MDA could have broader benefits against the major causes of childhood mortality, including diarrhoea, respiratory infections, and malaria.^9^, ^10^, ^11^, ^12^, ^13^ In 2009, a cluster-randomised trial reported a reduction in child mortality of approximately 50% after azithromycin MDA^14^ and, in 2018, stronger evidence was provided by the Mortality Reduction After Oral Azithromycin (MORDOR) study, which reported a 13·5% reduction in child mortality in azithromycin-treated communities compared with placebo in three African countries (Malawi, Niger, and Tanzania).^15^ Additional data analyses have considered the optimal timing and target population of azithromycin MDA for reducing child mortality^16^, ^17^ and guidelines were produced by WHO in 2020.^18^ However, the development of macrolide resistance, which has long been shown to occur after azithromycin MDA for trachoma control, is an important concern.^19^, ^20^, ^21^, ^22^ The development of macrolide resistance in nasopharyngeal *Streptococcus pneumoniae* has been reported for two of the MORDOR sites. In Niger, the prevalence of macrolide resistance was 12·8% in azithromycin communities at the 24-month follow-up, and 2·9% in placebo communities.^23^ At the 24-month follow-up in Tanzania, 13·4% of isolates were macrolide-resistant in azithromycin communities versus 13·2% in placebo communities.^24^

**Research in context**

**Evidence before this study**

We searched PubMed on March 28, 2021, using the search terms “azithromycin”, “resistance”, and “*Streptococcus pneumoniae*” and either “mass drug administration” or “trachoma”, and the variations “pneumococcus” and “mass treatment”. The search did not include restrictions for language or a start date. We included studies of azithromycin mass drug administration (MDA) that measured the prevalence of carriage of macrolide-resistant *S pneumoniae*. MDA of azithromycin to whole communities for control of trachoma generally produces an increase in macrolide resistance. The level and duration of macrolide resistance appears to be related to frequency of azithromycin distribution and baseline prevalence of resistance. However, very few studies have investigated the effect on macrolide resistance of azithromycin MDA targeted to children only. Data from the MORDOR study sites in Niger and Tanzania showed low rates of *S pneumoniae* and varying effects on macrolide resistance. At the Tanzania site, 6 months after the fourth and final biannual MDA, 13·4% of isolates were macrolide-resistant in azithromycin communities versus 13·2% in placebo communities. At the Niger site, at the same timepoint, macrolide resistance was 12·8% in azithromycin communities and 2·9% in placebo communities.

**Added value of this study**

In a large, cluster-randomised trial in Malawi, biannual azithromycin distributions to children aged 1–59 months significantly increased the proportion of *S pneumoniae* isolates resistant to macrolides. There was no evidence of cross-resistance to penicillin. The evidence from the other MORDOR sites, which had relatively low isolation rates of *S pneumoniae*, was conflicting as to whether azithromycin MDA targeted to children younger than 5 years increases macrolide resistance. This study provides evidence in a different setting that azithromycin MDA to reduce child mortality increases macrolide resistance.

**Implications of all the available evidence**

Evidence is increasing for a beneficial effect of azithromycin MDA on child mortality and WHO has released guidelines for when countries should consider this intervention. Results from the MORDOR study sites in Tanzania and Niger indicate increased macrolide resistance in the setting of azithromycin MDA in Niger but not in Tanzania. The results of this study emphasise the importance of careful monitoring of macrolide resistance, and the need for further research into its implications in low-income settings, as the intervention is implemented more widely.

The development of macrolide resistance in *C trachomatis* has not been reported as a serious concern after azithromycin MDA, although resistance at extra-ocular sites, especially nasopharyngeal carriage of *S pneumoniae*, might be of greater concern. One third of *S pneumoniae* strains worldwide have been estimated to be macrolide resistant.^25^ Because antibiotic resistance in a community appears to correlate with the volume of antibiotic given, an understanding of the effects of azithromycin MDA for reducing child mortality on antibiotic resistance, particularly in *S pneumoniae*, will be essential for countries to formulate health policies relating to this intervention.^26^ To investigate antibiotic resistance after azithromycin MDA for reducing child mortality, nasopharyngeal swabs were collected from children in randomly selected communities at the MORDOR Malawi study site. We report nasopharyngeal *S pneumoniae* carriage and macrolide and penicillin resistance before and after biannual azithromycin MDA in children aged 1–59 months in Malawi. The main outcomes are assessed at the individual level, accounting for clustering by community. This was a preconceived cluster-randomised controlled trial conducted within MORDOR.

## Methods

### Study design

This study was performed as part of the MORDOR trial in Mangochi District, Malawi. Before MORDOR commenced, 30 communities were randomly selected from all eligible communities in Mangochi District for surveillance of morbidity outcomes. Allocation of communities to either morbidity assessment or the MORDOR mortality outcome was performed at the coordinating centre for the study at the University of California, San Francisco (CA, USA). The randomisation unit (community) was defined as the catchment area of a health surveillance assistant, which usually has a total population of approximately 1000. Communities with a total population greater than 2000 on a pre-baseline study census were excluded. The randomisation was restricted to six communities in each of the five administrative zones of Mangochi District (Makanjira, Namwera, Chilipa, Monkey Bay, and Mangochi) for logistical reasons and geographical generalisability. Biannual community visits were conducted as for the MORDOR study to record census updates, household GPS coordinates, and administration of the study drug, which has previously been described in detail.^15^

Ethical approval for this study was obtained from the ethics committees at the College of Medicine, University of Malawi, Blantyre, and the London School of Hygiene & Tropical Medicine, London, UK.

### Participants

Participants were selected from all children younger than 5 years identified in the study clusters at the biannual census visits who had slept at the household the previous night. Individuals were removed or added to the cohort at each of the biannual follow-up censuses depending on age and residence status. All children aged 1–59 months and weighing at least 3·8 kg were eligible for treatment biannually for a total of four distributions. Only children allergic to macrolides or azalides were not offered treatment. At the baseline, 12-month, and 24-month follow-up visits, a sample of 40 children per community, aged 1–59 months, were randomly selected from all censused children using a function of the data collection app, to undergo nasopharyngeal swabbing (target of 1200 swabs at each visit). Parents or guardians provided written informed consent for tests after discussion with the study nursing staff speaking the local language. Parents or guardians who were illiterate provided a thumb print to acknowledge consent.

Multiple visits by study supervisors, fieldworkers, and nurses were made to study villages before and during the study for sensitisation, discussion of research conduct, and to plan dissemination of findings. Study findings were discussed at community meetings across the study site.

### Randomisation and masking

Placebo and azithromycin (both donated by Pfizer, New York, NY, USA) were provided with identical labels to ensure blinding. Six letters were used by the manufacturer to label the study drug, three of which corresponded to azithromycin and three to placebo. Communities were randomly assigned to a drug letter using the sample function in R software, version 3.1. Simple randomisation without stratification was performed at the coordinating centre for the study at the University of California, San Francisco. All study staff and participants in Malawi were masked to the treatment code until after all field work and data collection were complete.

### Procedures

Azithromycin 20 mg/kg or placebo were administered as an oral suspension biannually for a total of four treatments. The placebo contained the vehicle of the oral azithromycin suspension. Children who were able to stand received an approximate dose based on their height, measured using a height–dose stick, and smaller children were weighed. The height–dose stick was optimised using local anthropometry data from the study site. Distribution of the drug was done after sample collection was complete and was performed by the health surveillance assistants and fieldworkers conducting house-to-house visits. Parents or guardians were asked to inform the health surveillance assistant of any adverse events that occurred within 7 days of receiving the study drug. Health surveillance assistants subsequently informed the study team.

Sample collection was done during the baseline visit (May to July, 2015), 12-month follow-up (April to June, 2016), and 24-month follow-up (March to June, 2017). A nasopharyngeal swab sample was collected via the nasal passage from selected children using a FLOQSwab (Copan Diagnostics, Murrieta, CA, USA). Sample tubes were labelled with a random number and barcode and scanned using a feature of a custom-built data collection application (Conexus, Salt Lake City, UT, USA) on Android devices to link to census data. Samples were stored in skim milk tryptone glucose glycerine medium and placed on ice in the field. Samples were then frozen at −80°C at Mangochi District Hospital each afternoon. Samples were transported regularly, on ice packs, to Blantyre, where microbiological testing took place at Malawi-Liverpool-Wellcome Trust Laboratories.

For microbiological processing, samples were thawed at room temperature and 10 μL of the transport medium was inoculated onto gentamicin blood agar plates using a calibrated loop. An optochin disc (5 μg) was placed near the initial inoculum and plates were incubated for 24 h at 37°C in 5% carbon dioxide. *S pneumoniae* was identified as optochin-susceptible, α-haemolytic colonies. A single, well-isolated colony was re-streaked onto blood agar and grown overnight under the same culture conditions. Azithromycin and penicillin sensitivity of the purified isolate was then tested using the Kirby-Bauer method with ISO-blood agar, 15 μg azithromycin discs, and 1 μg oxacillin discs. Zones of inhibition were measured and scored the next day according to M100 Clinical and Laboratory Standard Institute guidelines.^27^ Isolates were defined as azithromycin resistant if the azithromycin disc zone diameter was 13 mm or less, and penicillin resistant if the oxacillin disc zone diameter was 19 mm or less.

### Outcomes

The primary prespecified outcome was the proportion of *S pneumoniae* isolates exhibiting macrolide resistance in children aged 1–59 months at the 12-month follow-up visit (6 months after the second biannual treatment visit) and the 24-month follow-up visit (6 months after the fourth biannual treatment visit). Prespecified secondary outcomes were carriage rates of *S pneumoniae* and the proportion of *S pneumoniae* isolates resistant to penicillin at 12 months and 24 months. The following prespecified secondary outcomes assessed in the MORDOR study in Malawi have been published elsewhere: malaria parasitaemia and haemoglobin;^28^ cost-effectiveness of the intervention for reducing mortality;^6^ and cause-specific mortality rates.^29^ The samples have not yet been processed to assess outcomes related to prevalence of macrolide resistance in stool samples and the fraction of conjunctival swabs yielding ocular chlamydia.

### Statistical analysis

600 children per treatment group were selected for sampling at each round (40 children from 15 communities in each group), standardised across the three MORDOR country sites. In Malawi, using a conservative prediction of pneumococcal carriage rates of 40% in preschool children, we expected 240 isolates to be cultured in the placebo group at each time point.^30^ This would provide approximately 80% power to detect a 20% increase in nasopharyngeal pneumococcal macrolide resistance from a baseline of 12% in the azithromycin-treated group compared with the placebo group, assuming an α of 0·05 and an intra-cluster correlation coefficient of 0·11 for pneumococcal carriage.^20^

In the intention-to-treat population, prevalence of *S pneumoniae*, resistance of strains to macrolides, and resistance of strains to penicillin were assessed by treatment group at the individual level at the 12-month and 24-month follow-up visits using mixed-effects logistic regression models, including random effects for randomisation unit and fixed effects for baseline values for *S pneumoniae* carriage, macrolide resistance, and penicillin resistance. Odds ratios and respective 95% CIs are presented for the proportion of individuals carrying *S pneumoniae*, and the proportion of *S pneumoniae* strains resistant to macrolides and penicillin, in the azithromycin group compared with placebo. In addition, per-protocol analyses were performed using similar mixed-effects logistic regression models to assess the effect of treatment on *S pneumoniae* carriage, macrolide resistance, and penicillin resistance. The per-protocol analyses included only children who received the assigned treatment at the previous visit (ie, the 6-month treatment round for the 12-month follow-up visit and the 18-month treatment visit for the 24-month follow-up visit). Age and sex were not included as fixed effects because they were balanced at baseline and sensitivity analyses including these covariates showed minimal change in the outcomes. All analyses were conducted using Stata version 15.

Mean *S pneumoniae* carriage, mean proportion of strains resistant to azithromycin, and mean proportion of strains resistant to penicillin at the 12-month and 24-month visits, were also assessed at the community level by treatment group using a generalised linear model. No adjustment was made for baseline prevalence of *S pneumoniae*, macrolide resistance, and penicillin resistance, which were similar between groups. Given the sampling method of 40 children per community, very few siblings were selected; therefore, family clustering bias would not affect the results.

The baseline prevalence of azithromycin resistance was displayed geographically using QGIS software (version 3.4.13). Inverse distance weighting interpolation was used to create a smooth surface of estimated macrolide resistance around the 30 clusters where samples were collected.

This study is registered with ClinicalTrials.gov, NCT02048007.

### Role of the funding source

The funder of the study had no role in study design, data collection, data analysis, data interpretation, or writing of the report.

## Results

**Figure 1:**
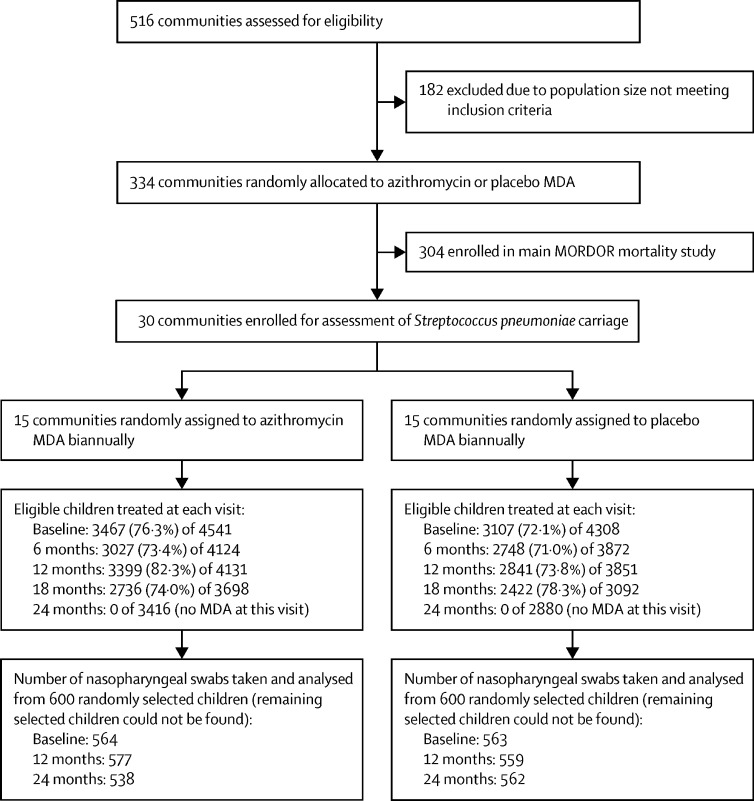
Trial profile MDA=mass drug administration.

At the 12-month visit (April 15 to June 9, 2016), 4131 children were eligible for sampling in the azithromycin group and 3851 children were eligible in the placebo group. At 24 months (March 16 to June 29, 2017), 3416 children were eligible in the azithromycin group and 2880 in the placebo group. Data from the 15 communities in each group are included in all analyses. Treatment coverage over the four rounds of the study was 76·6% in azithromycin communities (12 629 treatments administered to 16 494 eligible children) and 73·5% in placebo communities (11 118 treatments administered to 15 123 eligible children); detailed breakdown by treatment round is shown in figure 1. The number of nasopharyngeal swabs included in the analysis from 600 children ranged from 538 to 577 in the azithromycin communities and from 559 to 563 in the placebo communities (figure 1). No serious adverse events attributable to the study drug were reported in the study.

**Figure 2:**
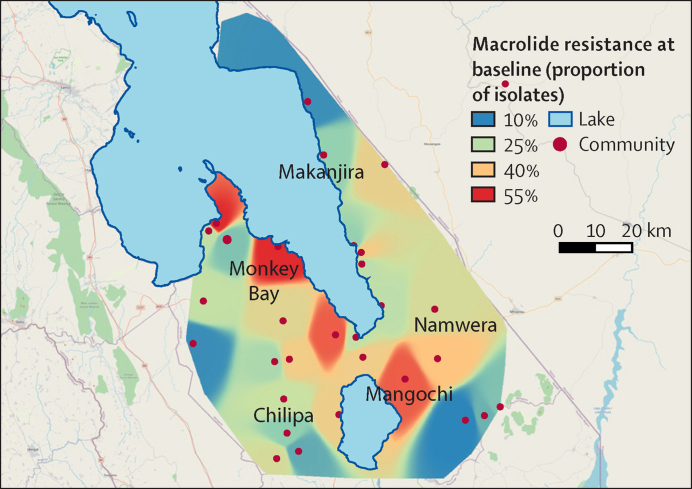
Distribution of macrolide resistance in Mangochi District at baseline Resistance is shown as a colour ramp, with intermediate percentages showing as shades of colour in between those shown in the legend.

Details of sampled children at the baseline visit are shown in table 1. Age and sex distributions were similar between azithromycin and placebo communities. These characteristics were similarly balanced at 12 months and 24 months (data not shown). Baseline carriage of *S pneumoniae* was greater than 85% in both groups. Macrolide resistance was present in 28% of isolates in both groups at baseline (136 of 481 isolates in the placebo group and 135 of 489 isolates in the azithromycin group) and penicillin resistance was present in approximately 45% of isolates (216 of 481 isolates in the placebo group and 228 of 489 isolates in the azithromycin group; table 1). Prevalence of macrolide resistance at baseline is shown on the map of Mangochi District (figure 2). A higher proportion of isolates showed resistance around Mangochi town and along the main transport and tourist road to Monkey Bay than elsewhere in the District. Resistance was also quite high to the east, towards the Mozambique border, and lower in more remote parts of the Makanjira, Namwera, Chilipa, and Monkey Bay zones.

**Table 1:** Baseline characteristics of children selected for bacteriological sampling

		**Placebo group (n=563)**	**Azithromycin group (n=564)**
Sex			
	Female	300 (53%)	284 (50%)
	Male	263 (47%)	280 (50%)
Age group, months			
	1–11	86/558 (15%)	101/560 (18%)
	12–23	126/558 (23%)	126/560 (23%)
	24–35	116/558 (21%)	117/560 (21%)
	36–47	116/558 (21%)	113/560 (20%)
	48–59	114/558 (20%)	103/560 (18%)
*Streptococcus pneumoniae* carriage		481 (85%)	489 (87%)
	Macrolide resistance	136/481 (28%)	135/489 (28%)
	Penicillin resistance	216/481 (45%)	228/489 (47%)

Data are presented as n (%) or n/N (%).

In the intention-to-treat analysis, *S pneumoniae* carriage was similar between groups at the 12-month and 24-month follow-up visits. At both visits, macrolide resistance was higher in the azithromycin group compared with the placebo group, whereas the proportion of *S pneumoniae* strains showing penicillin resistance was similar in the two groups (table 2). The per-protocol analysis showed similar results to the intention-to-treat analysis (table 2), thus not providing any evidence for a difference in the effect of the intervention on *S pneumoniae* carriage, macrolide resistance, or penicillin resistance in those who actually received study drug at the previous MDA round, compared with the effect in the overall study population.

**Table 2:** *S pneumoniae* carriage, macrolide resistance, and penicillin resistance at the 12-month and 24-month follow-up visits

		**Proportion of individuals carrying** S pneumoniae				**Proportion of macrolide-resistant *S pneumoniae* isolates**				**Proportion of penicillin-resistant *S pneumoniae* isolates**			
		n/N	Proportion, % (95% CI)	OR (95% CI)*	p value	n/N	Proportion, % (95% CI)	OR (95% CI)*	p value	n/N	Proportion, % (95% CI)	OR (95% CI)*	p value
**12 months**													
Intention to treat													
	Placebo	450/558	80·6% (77·4–83·9)	1 (ref)	..	97/450	21·6% (17·7–25·4)	1 (ref)	..	173/450	38·4% (33·9–43·0)	1 (ref)	..
	Azithromycin	472/577	81·8% (78·6–85·0)	1·06 (0·69–1·62)	0·79	174/472	36·9% (32·5–41·2)	2·26 (1·46–3·49)	0·0002	200/472	42·4% (37·9–46·8)	1·16 (0·88–1·52)	0·29
Per protocol													
	Placebo	327/400	81·8% (77·9–85·6)	1 (ref)	..	74/327	22·6% (18·1–27·2)	1 (ref)	..	122/327	37·3% (32·0–42·6)	1 (ref)	..
	Azithromycin	350/421	83·1% (79·5–86·7)	1·10 (0·68–1·77)	0·71	141/350	40·3% (35·1–45·4)	2·47 (1·56–3·90)	0·0001	150/350	42·9% (37·6–48·1)	1·24 (0·89–1·72)	0·20
**24 months**													
Intention to treat													
	Placebo	457/562	81·3% (78·1–84·5)	1 (ref)	..	150/457	32·8% (28·5–37·1)	1 (ref)	..	210/457	46·0% (41·4–50·5)	1 (ref)	..
	Azithromycin	440/538	81·8% (78·5–85·1)	1·06 (0·67–1·68)	0·80	193/440	43·9% (39·2–48·5)	1·66 (1·15–2·40)	0·0069	173/440	39·3% (34·7–43·9)	0·74 (0·48–1·13)	0·16
Per protocol													
	Placebo	319/387	82·4% (78·6–86·2)	1 (ref)	..	110/319	34·5% (29·2–39·7)	1 (ref)	..	145/319	45·5% (40·0–50·9)	1 (ref)	..
	Azithromycin	294/362	81·2% (77·2–85·3)	0·90 (0·50–1·61)	0·72	126/294	42·9% (37·2–48·5)	1·52 (1·05–2·18)	0·025	110/294	37·4% (31·9–43·0)	0·69 (0·44–1·08)	0·11

Per-protocol analysis included only those who received the study drug at the previous visit (6 months earlier). *S pneumoniae=Streptococcus pneumoniae.* OR=odds ratio.

* OR from mixed-effects logistic regression, including randomisation unit as a random effect and baseline values for *S pneumoniae* carriage, azithromycin resistance, and penicillin resistance in the respective models.

The analysis of the effect of azithromycin at the community level indicated similar results to the individual-level analysis. Specifically, it showed similar *S pneumoniae* carriage and penicillin resistance between treatment groups and higher macrolide resistance in the azithromycin group (appendix p 1).

## Discussion

This study assessed the prevalence of *S pneumoniae* carriage, macrolide resistance, and penicillin resistance in the setting of the MORDOR study in Malawi. Communities (clusters) were randomly selected to produce data generalisable to the whole MORDOR Malawi study site (Mangochi District). The results indicate that carriage of *S pneumoniae* was not affected by up to four biannual rounds of azithromycin MDA in Malawi. Macrolide resistance, however, was higher in *S pneumoniae* isolates in azithromycin-treated communities compared with placebo-treated communities at the 12-month and 24-month follow-up visits. The difference in macrolide resistance between those who received azithromycin compared with placebo at the previous visit was not greater than that identified in the primary intention-to-treat analysis. This might be expected given the relatively high coverage of study drug (71–82% coverage per round) and given that *S pneumoniae* strains are frequently passed between individuals in a household or community, particularly by toddlers and older children.^22^ There was no increase in penicillin resistance in the azithromycin group, an important additional consideration due to the well-documented potential for development of cross-resistance in bacteria.^31^

Antibiotic resistance studies, mostly conducted in the setting of trachoma control, have generally reported an increase in resistance after azithromycin MDA. However, the extent of the increase in resistance appears to vary considerably, possibly related to background antibiotic use and previous azithromycin MDA. A 1997 study of Australian Aboriginal children younger than 15 years with trachoma, who were treated along with household contacts who were children, showed the carriage of resistant strains of *S pneumoniae* increased from 1·9% at baseline to 54·5% 2–3 weeks after treatment, then decreased to 34·5% 2 months after treatment, and 5·9% 6 months after treatment.^21^ These findings suggest that resistance wanes with time after treatment. Studies in Nepal and Tanzania have similarly indicated low levels of macrolide resistance 6–12 months after one or three rounds of azithromycin MDA, although *S pneumoniae* was only isolated from 7–12% of swabs in the Tanzanian study.^32^, ^33^, ^34^ A study in The Gambia, where there is little routine use of macrolides, reported carriage of macrolide-resistant *S pneumoniae* to be only 1·2% 1 month after three biannual rounds of azithromycin MDA, decreasing to 0·9% 6 months after MDA.^35^

High levels of macrolide resistance have been reported at more than a year of follow-up after several rounds of azithromycin MDA. A study in Ethiopia reported that 28·2% of *S pneumoniae* isolates were macrolide-resistant 6 months after four biannual MDA rounds, increasing to 76·8% 6 months after the sixth and final biannual MDA. The proportion of resistant isolates was 30·6% 12 months after the final MDA and 20·8% 24 months after, again suggesting that resistance decreases over time even from higher levels after multiple rounds of MDA.^36^ Background use of macrolides was reportedly low in the study area and resistance in neighbouring control communities was 0–0·9%, although many years of azithromycin MDA have been distributed for trachoma in Ethiopia. Another cluster-randomised study in Ethiopia reported an increase in macrolide resistance from 6·3% of isolates before treatment to 62·3% 3 months after four 3-monthly rounds of azithromycin MDA.^20^ Macrolide resistance in control communities was 11·6%.

In 2013, Coles and colleagues^19^ investigated the proportion of resistant isolates at several timepoints after a single round of azithromycin MDA in Tanzania.^19^ Before treatment, the proportion of resistant isolates was 2·1%, increasing to 4·5% at 1 month after MDA, 18·3% at 3 months, and 35·4% at 6 months. In communities that did not receive MDA, the proportion of resistant isolates was 13·1% before treatment, 4·4% at 1 month, 8·2% at 3 months, and 12·4% at 6 months. The increase in resistance at successive follow-up points in this study is unusual and difficult to explain, but it could be related to antibiotic use outside of MDA programmes. Indeed, more than 65% of people in the study communities reported taking drugs to treat suspected infection in the 30 days before sampling.

In 2015, a systematic review of antibiotic resistance in *S pneumoniae* after azithromycin distribution for trachoma identified that resistance prevalence was dependent on the frequency of azithromycin distribution and baseline prevalence of resistance.^37^ Resistance gradually decreased over time after the last distribution. It is possible that persistent macrolide resistance could occur above a certain threshold of macrolide use. This could become increasingly important clinically because routine use of macrolides might be needed as pneumococcal infections become more commonly penicillin-resistant. The data presented suggest that this persistance of resistance is not currently the case and that macrolide resistance is gradually eliminated, presumably due to a fitness cost of carrying macrolide resistance.

Baseline macrolide resistance was high in this study at 28% (as was penicillin resistance at 45%) but the proportions were similar to those found in clinical isolates in Blantyre (unpublished data). Macrolides are not used in frontline management of *S pneumoniae* syndromes in Malawi, although trachoma control programmes have conducted azithromycin MDA for several years in a small number of districts across Malawi.^38^ MDA for trachoma was not conducted in Mangochi but it is possible that some azithromycin might have been used outside of the MDA programme, and it is theoretically feasible that, because azithromycin is mainly excreted in the faeces unchanged, there could be environmental contamination of the waterways that also feed Lake Malawi. Mangochi also has a long and porous border with Mozambique, where antibiotics, particularly amoxicillin with clavulanic acid, azithromycin, and cotrimoxazole, are commonly available over the counter.^39^, ^40^ The distribution of macrolide resistance at baseline would be compatible with antibiotics being brought from Mozambique along the main road to Mangochi town and subsequently along the main transport and tourist road to Monkey Bay. The reasons for relatively high macrolide resistance at the MORDOR Malawi study site are not clear at this stage, although they are consistent with levels of resistance seen in clinical isolates.

The levels of resistance reported at the other MORDOR sites (up to 13·4%) are lower than at the Malawi site, and the reasons for this are not clear. The isolation rates of *S pneumoniae* were lower at the Niger and Tanzania sites than in Malawi: 54·7% in placebo communities and 54·0% in azithromycin communities at 24 months at the Niger site, and 11·0% in placebo communities and 15·1% in azithromycin communities at 24 months at the Tanzania site. It is feasible that any fitness cost of carrying macrolide resistance could lead to preferential isolation of non-resistant isolates, and that this would be exaggerated if isolation rates were low.

It is not clear what effect macrolide resistance might have on the efficacy of azithromycin MDA for reducing child mortality. Increased resistance could be assumed to decrease efficacy due to a diminished effect against resistant pathogens. However, there is currently no evidence to support this possibility. In fact, the MORDOR study showed an increase in the effect estimate with each successive follow-up period, despite the two sites where the effect was greatest, Niger and Malawi, also reporting an increase in macrolide resistance in the azithromycin communities. An increase in effect could be independent of antibiotic resistance, such as through a cumulative reduction in pathogens with each MDA, or directly related to the resistance, such as if the fitness cost to bacteria of carrying resistance reduced their virulence.^41^

A limitation of this study is that follow-up took place at 12-month intervals, 6 months after the previous MDA. More granular and longer-term follow-up would have been preferable but was not possible due to funding and logistical limitations. Resistance would probably have peaked at higher levels than those we measured in the weeks after MDA and reduced by the time of sampling. However, our findings show persistent resistance of at least 6 months' duration, which is an important consideration for policymakers and is consistent with other studies. Samples were also available at both the midpoint and endpoint of this study, enabling assessment of the change in antibiotic resistance with continued MDA. The results do not provide evidence that macrolide resistance increases further after four rounds compared with two rounds of biannual azithromycin MDA to children aged 1–59 months. This study did not investigate the development of resistance in other organisms, which could be of clinical significance and could serve as a reservoir of resistance genes. Finally, it was outside of the scope of this study to investigate the mechanisms of macrolide resistance via molecular detection of markers of resistance in this setting, which could form part of future research efforts.

The results of this study indicate that macrolide resistance in *S pneumoniae* increases after four biannual rounds of azithromycin MDA to children aged 1–59 months in Malawi. Previous research suggests that the development of macrolide resistance varies greatly by population and might increase further and become more persistent with additional rounds of MDA or if there is macrolide use in primary health care.^19^, ^36^ However, our understanding of the prevalence of macrolide resistance and its mechanisms in the setting of azithromycin MDA, particularly when targeted solely to children to reduce mortality, remains poor. Prediction of the resistance that would be generated from implementation in other regions and countries, if MDA were deployed more widely, would currently be extremely challenging. Further research into azithromycin resistance should remain a key component of studies and interventions with azithromycin MDA for the reduction of child mortality.

## Data sharing

Parents and guardians of participants in the MORDOR Malawi morbidity study consented to confidential use of the samples and data collected by the study team only; hence, wider availability of the data is not possible. The full MORDOR study protocol is available at https://clinicaltrials.gov/ct2/show/NCT02047981.

## Declaration of interests

We declare no competing interests.
