# Supplementary material for: Prevalence of nasopharyngeal Streptococcus pneumoniae carriage and resistance to macrolides in the setting of azithromycin mass drug administration: analysis from a cluster-randomised controlled trial in Malawi, 2015–17
Source: Lancet Microbe. 2022 Feb;3(2):e142–50. doi: 10.1016/S2666-5247(21)00279-2 (PMC8819720; doi:10.1016/S2666-5247(21)00279-2)
Supplement: Supplementary appendix [file mmc1.pdf]

THE LANCET
Microbe

Supplementary appendix

This appendix formed part of the original submission and has been peer reviewed. We post it as supplied by the authors.

Supplement to: Hart JD, Samikwa L, Meleke H, et al. Prevalence of nasopharyngeal *Streptococcus pneumoniae* carriage and resistance to macrolides in the setting of azithromycin mass drug administration: analysis from a cluster-randomised controlled trial in Malawi, 2015–17. *Lancet Microbe* 2022; published online Jan 13. https://doi.org/10.1016/S2666-5247(21)00279-2.

Supplementary table 1: Mean *S. pneumoniae* carriage, mean azithromycin resistance, and mean penicillin resistance at the 12-month and 24-month follow-up visits, assessed at the community level by treatment arm

	Mean *S. pneumoniae* carriage			Mean proportion of *S. pneumoniae* strains resistant to azithromycin			Mean proportion of *S. pneumoniae* strains resistant to penicillin		
	Proportion (95% CI)	*OR (95%CI)	*P*-value	Proportion (95% CI)	*OR (95%CI)	*P*-value	Proportion (95% CI)	*OR (95%CI)	*P*-value
12 months									
Placebo	79.7 (73.7-85.7)	1		21.0 (15.9-26.2)	1		37.6 (32.2-43.0)	1	
Azithromycin	81.6 (77.1-86.0)	1.12 (0.73-1.72)	0.59	38.2 (29.3-47.1)	2.32 (1.50-3.59)	0.0002	43.2 (37.3-49.2)	1.27 (0.94-1.71)	0.12
24 months									
Placebo	81.7 (76.6-86.7)	1		33.0 (26.9-39.0)	1		45.3 (36.3-54.3)	1	
Azithromycin	82.1 (76.4-87.9)	1.03 (0.65-1.64)	0.90	43.7 (35.7-51.7)	1.58 (1.08-2.31)	0.019	38.3 (31.2-45.4)	0.75 (0.49-1.15)	0.19

*OR from generalized linear models comparing community means
